# Plant E3 Ligases and Their Role in Abiotic Stress Response

**DOI:** 10.3390/cells11050890

**Published:** 2022-03-04

**Authors:** Raed Al-Saharin, Hanjo Hellmann, Sutton Mooney

**Affiliations:** 1 Department of Applied Biology, Tafila Technical University, At-Tafilah 66110, Jordan; 2 School of Biological Sciences, Washington State University, Pullman, WA 99163, USA; hellmann@wsu.edu (H.H.); suttonmooney@wsu.edu (S.M.)

**Keywords:** E3 ligase, abiotic stress, drought stress, salt stress, oxidative stress, temperature stress, heavy metal stress

## Abstract

Plants, as sessile organisms, have limited means to cope with environmental changes. Consequently, they have developed complex regulatory systems to ameliorate abiotic stresses im-posed by environmental changes. One such system is the ubiquitin proteasome pathway, which utilizes E3 ligases to target proteins for proteolytic degradation via the 26S proteasome. Plants ex-press a plethora of E3 ligases that are categorized into four major groups depending on their structure. They are involved in many biological and developmental processes in plants, such as DNA repair, photomorphogenesis, phytohormones signaling, and biotic stress. Moreover, many E3 ligase targets are proteins involved in abiotic stress responses, such as salt, drought, heat, and cold. In this review, we will provide a comprehensive overview of E3 ligases and their substrates that have been connected with abiotic stress in order to illustrate the diversity and complexity of how this pathway enables plant survival under stress conditions.

## 1. Introduction

Worldwide agricultural production has been and continues to experience severe yield losses due to exposure to abiotic stress conditions, such as drought, salt, or heat. It has been shown in a recent study that 50% of worldwide crop production is lost as a result of abiotic stresses in which temperature, drought, and salt stresses contribute to more than 90% of such loss [1]. Moreover, the worldwide cost of climate change for crops is estimated to rise up to $80 billion at 2 °C warming per year [2].

Abiotic stress is essentially any environmental condition that negatively affects the growth and general development of a plant [3]. This may range from mechanical disturbances, such as strong winds, to rapid changes in light conditions on a cloudy day, but generally it is mostly connected with severe stress conditions, such as soil salinity, heat waves, and drought stress [3,4,5]. The sessile lifestyle of plants makes it impossible for them to avoid these stressors by simply changing their location; hence, they need to have strategies in place to rapidly respond to changes in their environment in order to survive and sustain their growth.

**Figure 1:**
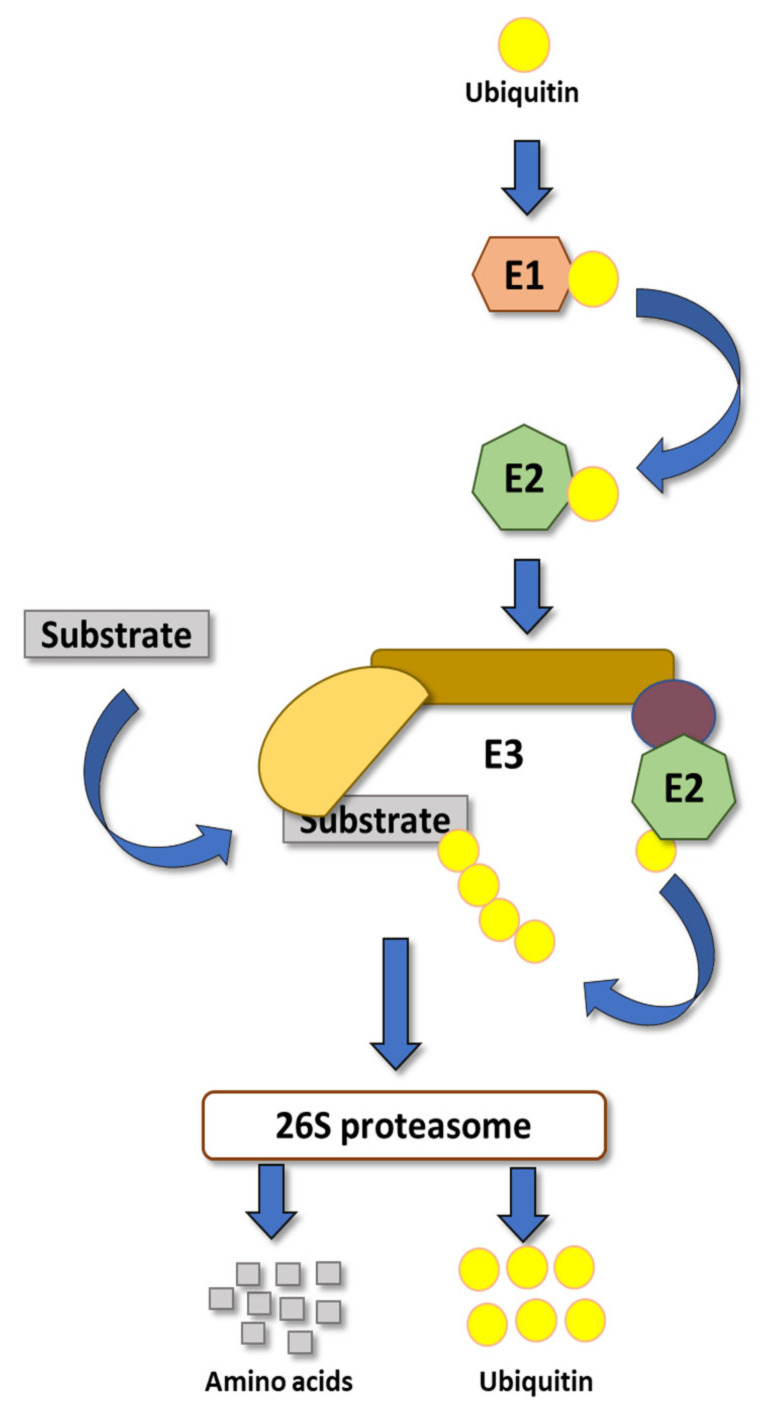
The ubiquitin proteasome pathway. Ubiquitin binds to E1, and E1 transfers the ubiquitin to E2, which binds with E3. E3 facilitates the transfer of ubiquitin from E2 to the substrate. Once the substrate is polyubiquitinated, it becomes a target for 26S proteasome for subsequent degradation.

An example that allows plants to respond to a very broad range of different stressors within minutes is represented by the ubiquitin proteasome pathway (UPP) [6]. The UPP is highly conserved among eukaryotes and mechanically comprises four basic steps (Figure 1). The first step is an ATP-dependent activation of ubiquitin (UBQ) by an E1 enzyme. The E1 binds the UBQ and transfers it in a second step to an UBQ-conjugating E2 enzyme. In the third step the E2 physically associates with an E3 UBQ ligase that has recruited a substrate protein to facilitate its ubiquitylation. Classically, build-up of a UBQ chain on the substrate marks the protein for the final step, which is degradation by the 26S proteasome (Figure 1) [7].

Plant genomes encode only a few E1 and E2 enzymes, while they have an abundance of E3s. For example, *Arabidopsis thaliana* and rice (*Oryza sativa*) have two and six E1s [8,9], respectively, 37 and 39 E2s [10,11], but more than 1100 predicted E3s [12,13]. Plant E3s can be classified into four major families based on their specific domains and complex formation: U-box, Homology to the E6-associated protein C-terminus (HECT) E3s, and really interesting new gene (RING)-finger, which are classified as either monomeric or multimeric E3 ligases [6]. 

The most diverse group within these E3s are multimeric E3 ligases that contain a cullin as their central scaffolding subunit. Here, the cullin interacts at its C-terminal region to a RING-finger domain containing protein [14]. The RING finger is a Zinc-binding domain that also facilitates E2 attachment [15]. The N-terminal region of the cullin interacts with substrate receptors that bring proteins into proximity with the cullin-bound E2 for ubiquitylation [16]. Four different cullin-based E3s have been described in plants. First, Skip1-Cullin1-F-box (SCF) complexes use proteins as substrate adaptors that contain an F-box motif [17,18]. SCF complexes have been widely described in context with development, light and phytohormone signaling, mitotic processes, and abiotic stress [14,19,20,21,22,23,24,25,26,27,28]. This wide range of SCF functions is not surprising since some plants can encode for more than 1000 F-box proteins [29,30]. Second, Cullin3-RING E3 ligases (CRL3) use proteins as substrate adaptors that contain a Broad-complex, Tramtrack, and Bric-à-brac/POxvirus and Zinc finger (BTB/POZ) domain [31,32]. BTB/POZ proteins are less abundant than F-box proteins, but can also vary between 80 to 100+ in plants [33,34,35]. CRL3s have been implicated with abiotic and biotic stress responses, as well as blue and red light signaling, flowering time, and metabolic processes [31,32,36,37,38,39,40,41,42,43]. Third, Cullin4 RING E3 ligases (CRL4) use proteins with a modified WD40 domain as substrate adaptors [44], and these adaptors number in the range of 100 in plants [45,46]. This class of E3 ligases is known for DNA damage and abiotic stress responses, as well as photomorphogenesis [47,48,49]. Finally, the Anaphase Promoting Complex/Cyclosome (APC/C) is required for sister chromatid separation in anaphase, and DNA replication processes in S-phase, but it also has been brought into context with auxin signaling [50,51]. 

Monomeric RING E3s facilitate substrate and E2 binding as a single moiety. They are highly diverse, and plants often encode for more than 400–500 different members [52]. Due to this diversity, they have been described in various developmental plant processes, but are also often involved in abiotic and biotic stress responses [6].

U-box E3s contain a modified RING finger domain that lacks the Zinc binding, but is still able to recruit E2s [53]. These E3 ligases also function as monomeric E3s and encompass less than 100 members in plants [54,55,56,57]. They function widely in abiotic and biotic stress responses, but have also been described, for example, in apical meristem maintenance or phytohormone signaling [58,59,60,61,62,63,64,65,66,67].

Finally, HECT domain E3s comprise the group with the fewest members in a given plant species, normally less than 20 [68,69,70]. They are monomeric E3s as well, and they have a distinct mechanism of ubiquitylation because they indirectly transfer the UBQ from the E2 to a substrate. The UBQ is first attached to a conserved lysine residue within the HECT E3 and then conjugated to a substrate [71]. HECT E3 are known for only a few biological processes, such as biotic stress, trichome development, and leaf senescence [70,72]. 

In the following, we will focus on the main abiotic stress conditions for crop plants, which negatively impact yield worldwide. In this context, we provide examples for E3 ligases for each of these stressors, and how they are thought to impact plant stress tolerance and stress responses. These stress-specific examples can be taken as an opportunity to target specific steps in the cellular network controlling abiotic stress responses. This can lead to the development of novel strategies for bioengineering resilient crop plants, with the aim of securing food production through sustainable agriculture in the upcoming years.

## 2. Drought Stress

Drought stress is one of the most widespread abiotic stresses that negatively affects crop productivity [73]. A recent study has shown that the reduction of crop yield due to drought stress ranges between 30–90%, depending on the crop species [74]. Drought arises when the amount of water in the soil is decreased and/or environmental conditions provoke continuous loss of water via transpiration or evaporation [75]. In the past years, E3 ligases have been established as important regulators of the drought stress response (Table 1), which we illustrate in several examples.

**Figure 2:**
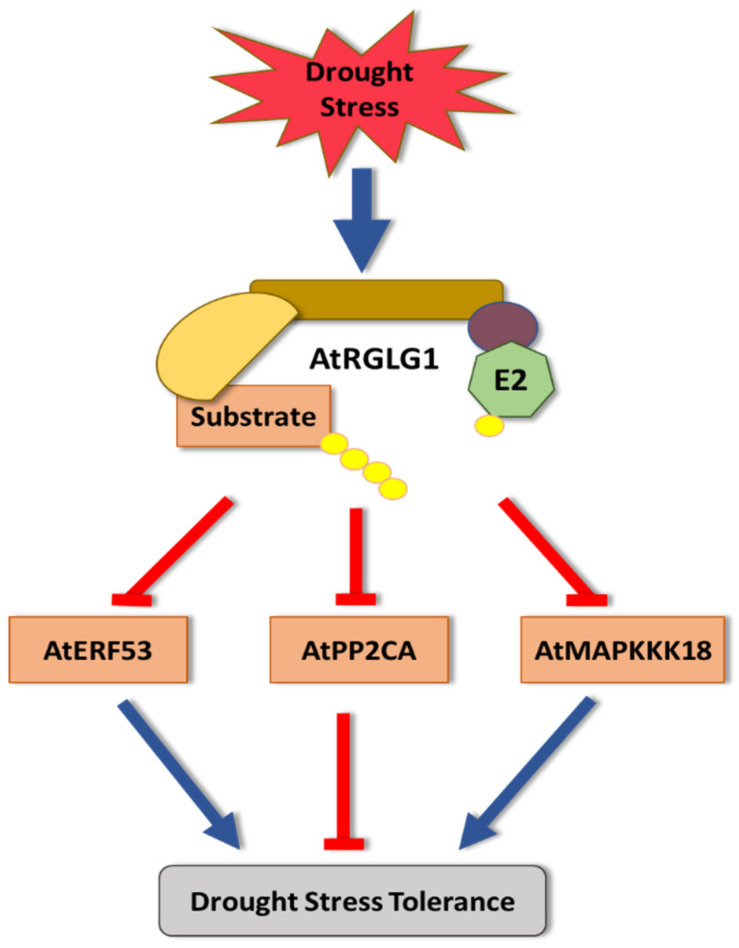
The role of *A. thaliana RING DOMAIN LIGASE1* (AtRGLG1) in drought stress tolerance. AtRGLG1 targets three proteins critical for improving drought tolerance for proteasomal degradation: ETHYLENE RESPONSE FACTOR53 (AtERF53), MITOGEN ACTIVATED PROTEIN KINASE KINASE KINASE 18 (MAPKKK18), and PROTEIN PHOSPHATASE 2CA (PP2CA).

Work on the *A. thaliana RING DOMAIN LIGASE1* (AtRGLG1) has demonstrated its role in targeting and promoting degradation of three key substrates: ETHYLENE RESPONSE FACTOR53 (AtERF53), MITOGEN ACTIVATED PROTEIN KINASE KINASE KINASE 18 (MAPKKK18), and PROTEIN PHOSPHATASE 2CA (PP2CA) [76,163,164] (Figure 2). AtERF53 is a transcription factor that belongs to the AP2/ERF transcription factor family [165], and MAPKKK18 is a member of the MAPKKKs family, which includes 80 members in Arabidopsis. It plays a role in senescence regulation, abiotic stress signaling, and ABA responses [166,167].

*Atmapkkk18* knockout transgenic lines have impaired stomatal closure and exhibit hypersensitivity toward drought stress. Transgenic lines overexpressing *AtERF53* or *AtMAPKKK18* in Arabidopsis showed increased drought tolerance compared to wildtype plants [76]. It has been shown that both RGLG1, and its closest sequelog RGLG2, negatively regulate drought stress tolerance by targeting and mediating the degradation of AtERF53 and AtMAPKKK18 [76,163]. In agreement with that, *rglg1/rglg2* double mutant lines showed increased AtERF53 and AtMAPKKK18 stability, and enhanced drought tolerance [167]. Additionally, even more robust drought tolerance was obtained when *MAPKKK18* or *AtERF53* were overexpressed in the *rglg1/rglg2* background. [163,165].

The third AtRGLG1 substrate, PP2CA, acts as a negative regulator of abscisic acid (ABA) signaling and ABA- dependent drought stress tolerance [168]. RGLG1 functions as a positive ABA response regulator by mediating PP2CA protein degradation, which then promotes ABA-mediated drought tolerance [164,169].

It seems that RGLG1 plays opposite roles in drought stress tolerance. It acts as a negative regulator by targeting both ERF53 and MAPKKK18, but functions as a positive regulator by targeting PP2CA (Figure 2). While this indicates the important role that RGLG1 plays in balancing the response toward drought stress, the mechanisms in which it does that are still not understood. 

Another RING finger E3 ligase in Arabidopsis and wheat (*Triticum aestivum*) that positively regulates drought stress tolerance is STRESS-ASSOCIATED PROTEIN 5 (TaSAP5), which mediates the degradation of DREB2A INTERACTING PROTEIN 1 (DRIP1) and DRIP2 [144]. DRIPs are RING finger E3 ligases that function as negative regulators of the drought stress response by facilitating degradation of DEHYDRATION-RESPONSIVE ELEMENT BINDING PROTEIN 2A (DREB2A) [170], an ERF/AP2 transcription factor known as a key regulator for drought and heat stress tolerance [28,171]. Overexpression of *TaSAP5* enhances the stability of DREB2A, which in turn upregulates drought-responsive genes, and improves drought tolerance in transgenic Arabidopsis and wheat plants [144].

In Arabidopsis, the expression of the F-box protein DROUGHT TOLERANCE RE-PRESSOR 1 (DOR1), a subunit of SKP1, CULLIN1, F-BOX (SCF) E3 ligase, was down-regulated under both drought stress treatment and exogenous ABA application [77]. *dor1* mutant lines are hypersensitive toward ABA, leading to stomatal closure, providing increased drought tolerance. On the other hand, overexpression of *DOR1* leads to increased drought sensitivity, indicating that DOR1 act as a negative regulator of drought stress tolerance [77].

**Figure 3:**
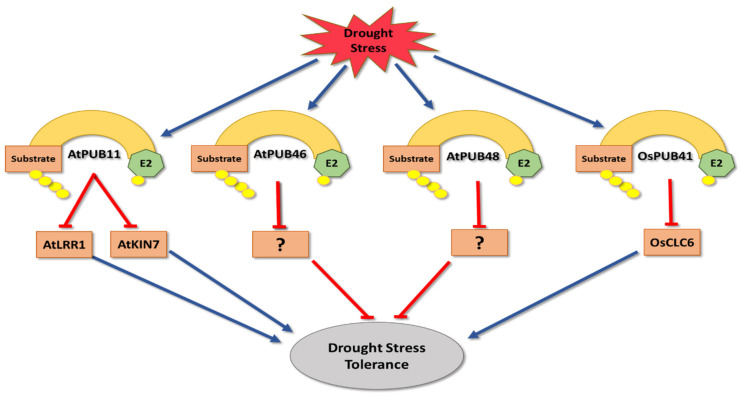
The role of PLANT U-BOX E3 ligases (PUBs) in drought stress tolerance. Both *A. thaliana* PUB46 and PUB48 positively regulate drought tolerance by targeting unknown substrates, which are suggested to be negative regulators of drought tolerance. On the other hand, AtPUB11 negatively regulates drought tolerance by targeting both LEUCINE RICH REPEAT PROTEIN 1 (LRR1) and KINASE 7 (KIN7), which are known as positive regulators of drought tolerance. Also, *O. sativa* PUB41 is considered to be a negative regulator of drought tolerance by targeting CHLORIDE CHANNEL 6 (OsCLC6).

Many members of PLANT U BOX (PUB) E3 ligases have been shown to be involved in drought stress tolerance regulation (Table 1). In Arabidopsis, PUB11 negatively regulates drought stress response by targeting LEUCINE RICH REPEAT PROTEIN 1 (LRR1) and KINASE 7 (KIN7) for degradation via the 26S proteasome [67]. Both LRR1 and KIN7 are needed to promote stomatal closure during water stress conditions [172,173]. On the other hand, Arabidopsis PUB46 and PUB48 act as positive regulators of drought stress since *pub46/pub48* double mutants become drought hypersensitive [93]. Although there is currently no information on their target substrates, it can be expected that they negatively regulate drought stress responses [93] (Figure 3).

In rice, OsPUB41 has been described as a negative regulator of drought stress tolerance. This E3 ligase is highly expressed under drought conditions, and compared to wildtype, *ospub41* null mutants have enhanced tolerance towards water deficit stress [65]. OsPUB41 triggers the degradation of CHLORIDE CHANNEL 6 (OsCLC6), a protein critical for drought stress tolerance [65], and part of a family that functions in modifying chlorine ion homeostasis under drought conditions [174] (Figure 3).

From stomatal closure to ABA response and chlorine ion homeostasis, E3 ligases have an important role in the regulation of drought response in plants. This section, with the selected examples, clearly demonstrates the relevance of plant E3 ligases in both positive and negative regulation of drought stress tolerance. It further emphasizes that these E3 ligases may represent critical tools to produce more resilient crop plants that better withstand drought stress in the future.

## 3. Salt Stress

Salinity in irrigation water sources and soil is a major environmental challenge due to the negative effects on plant growth, development, and productivity, especially in arid and semi-arid regions [175]. In the Middle East, more than 100 million hectares, around 6% of the total land area, consist of salinized soil or carries high sodium levels [176]. Globally, around 69% of wheat production is threatened by the negative influence of soil salinity [176]. Over the last two decades this issue has worsened due to the rising need to irrigate continually expanding arid regions, which often increases salt content in treated fields over time [177].

Under salt stress, several physiological and biochemical mechanisms are activated in plants, which can impact photosynthesis, distribution of harmful ions, and other biochemical adaptations [178,179]. It has been shown that several E3 ligases are involved in regulating these responses by targeting and mediating the degradation of salt stress-related proteins (Table 1).

In Arabidopsis, loss-of-function mutants affected in a plasma membrane associated RING finger E3 ligase called SALT TOLERANCE RING FINGER 1 (STRF1) displayed enhanced salt stress tolerance and decreased reactive oxygen species (ROS) accumulation. Although proteins targeted by this E3 ligase are still unknown, these findings indicate that STRF1 is a negative regulator of salt stress responses [102]. 

**Figure 4:**
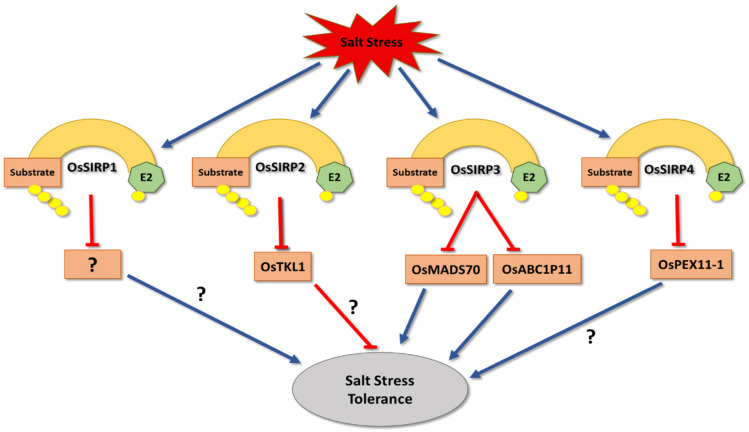
The role of *O. sativa* SALT-INDUCED RING PROTEIN family (SIRPs) in salt stress tolerance. OsSIRP1 negatively regulates salt tolerance by targeting unknown substrates that are suggested to be positive regulators of salt tolerance. Also, OsSIRP3 negatively regulates salt tolerance by triggering degradation of two salt-induced proteins, *O. sativa* MADS-BOX GENE 70 (OsMADS70) and an ABC DOMAIN CONTAINING PROTEIN (OsABC1P11). Moreover, OsSIRP4 is a negative regulator of salt stress tolerance by targeting *O. sativa* PEROXISOMAL BIOGENESIS FACTOR 11-1 (OsPEX11-1), which has an unknown function related to salt stress regulation. On the other hand, OsSIRP*2* is considered to be a positive regulator of salt tolerance by targeting its only known target substrate, TRANSKETOLASE 1 (OsTKL1).

Rice expresses many RING finger E3 ligases that are involved in salt stress regulation, such as the SALT-INDUCED RING PROTEIN family (SIRP) [123,124,125,126] (Figure 4). Three of the four members have been described as negative regulators of salinity stress responses [123]. Overexpression of *OsSIRP1* in Arabidopsis showed a reduction in salinity tolerance during seed germination and root elongation growth, which is a likely impact of the degradation of its still undefined target proteins [123]. Similarly, plants overexpressing *OsSIRP3* and *OsSIRP4* exhibit hypersensitivity to salt stress conditions [125,126]. OsSIRP3 accomplishes this by triggering degradation of two salt-induced substrate proteins, *O. sativa* MADS-BOX GENE 70 (OsMADS70) and an ABC DOMAIN CONTAINING PROTEIN (OsABC1P11) [125]. OsSIRP4 regulates the degradation of *O. sativa* PEROXISOMAL BIOGENESIS FACTOR 11-1 (OsPEX11-1) by the 26S proteasome [126]. However, it is currently unclear how exactly salt stress tolerance is affected by the OsSIRP4/OsPEX11-1 interaction [126].

On the other hand, overexpression of *OsSIRP2* E3 ligase enhances resistance towards salt and osmotic stress in Arabidopsis [124]. Yeast two-hybrid (Y2H) and pulldown assays have demonstrated physical interaction between OsSIRP2 and a potential target, *O. sativa* TRANSKETOLASE 1 (OsTKL*1*) [124]. OsTKL1 belongs to the transketolase family involved in the regeneration of ribulose 1,5-bisphosphate within the Calvin cycle [180,181], but it remains an open question as to how its ubiquitination and degradation affects salt stress responses [124].

As mentioned, four SIRP E3 ligases have been described to be involved in positive or negative regulation of salt stress tolerance. Although their mechanism of action is still unknown, they are good examples of E3 ligases that could be targeted for genetic modification to improve salinity tolerance in rice.

In cotton (*Gossypium hirsutum*), the RING finger E3 ligase SALT-ASSOCIATED RING FINGER PROTEIN (GhSARP1) is described as a negative regulator of salt stress tolerance [156]. When overexpressed in Arabidopsis, GhSARP1 enhanced salt stress sensitivity in transgenic plants during germination and seedling stages [156].

Currently, little is known about the role of PLANT U-BOX (PUB) E3 ligases in abiotic stress tolerance in wheat. Recently TaPUB15 was described as a positive regulator of salt stress tolerance, since overexpression of *TaPUB15* in both *Arabidopsis* and rice increased salt stress tolerance [142]. The transgenic lines showed deeper and more branched root development along with improved maintenance of low Na^+^/K^+^ ratios under salt stress when compared to wild type. Targets of TaPUB15 are currently unknown [142].

In contrast, TaPUB26 acts as a negative regulator of salt stress tolerance [143]. Here, stiff brome (*Brachypodium distachyon*) transgenic lines overexpressing this E3 ligase exhibited higher sensitivity toward salinity, which was accompanied by accumulation of ROS, and an increased Na^+^/K^+^ ratio [143]. TaPUB26 interacts with *T. aestivum* REGULATORY PARTICLE AAA-ATPASE 2A (TaRPT2a), which functions as an ATPase subunit of the 26S proteasome complex. Its gene expression is salt inducible, however the mechanism of how TaPUB26-TaRPT2a interaction affects salt stress tolerance is still not understood [143].

With increasing arid land that will require expanded irrigation systems, understanding how to address the impacts of salt on agriculture has become quite relevant. Work across a diverse group of plants underscores that E3 ligases are central players in how pathways are controlled when higher salt levels are present. Identification of substrates degraded by RING-finger and PUB E3s that are linked to salt stress response is providing some possible mechanisms that could be modulated to allow continued farming in areas like the Middle East, which will continue to see diminishing arable fields.

## 4. Oxidative Stress

Although oxygen (O_2_) is a fundamental element for land plants to obtain their energy resources by cellular respiration; it generates another challenge through the formation of ROS, such as superoxide (O_2_^−^), hydrogen peroxide (H_2_O_2_), or hydroxyl radicals (OH) [182]. ROS generally affect plant growth and development negatively via protein oxidation, lipid peroxidation, nucleic acid damage, enzyme deactivation, and cell death enhancement [183]. The rise in ROS can be observed by prolonged exposure to any abiotic stress, independent of whether it is e.g., salt, drought, or excessive light [184].

**Figure 5:**
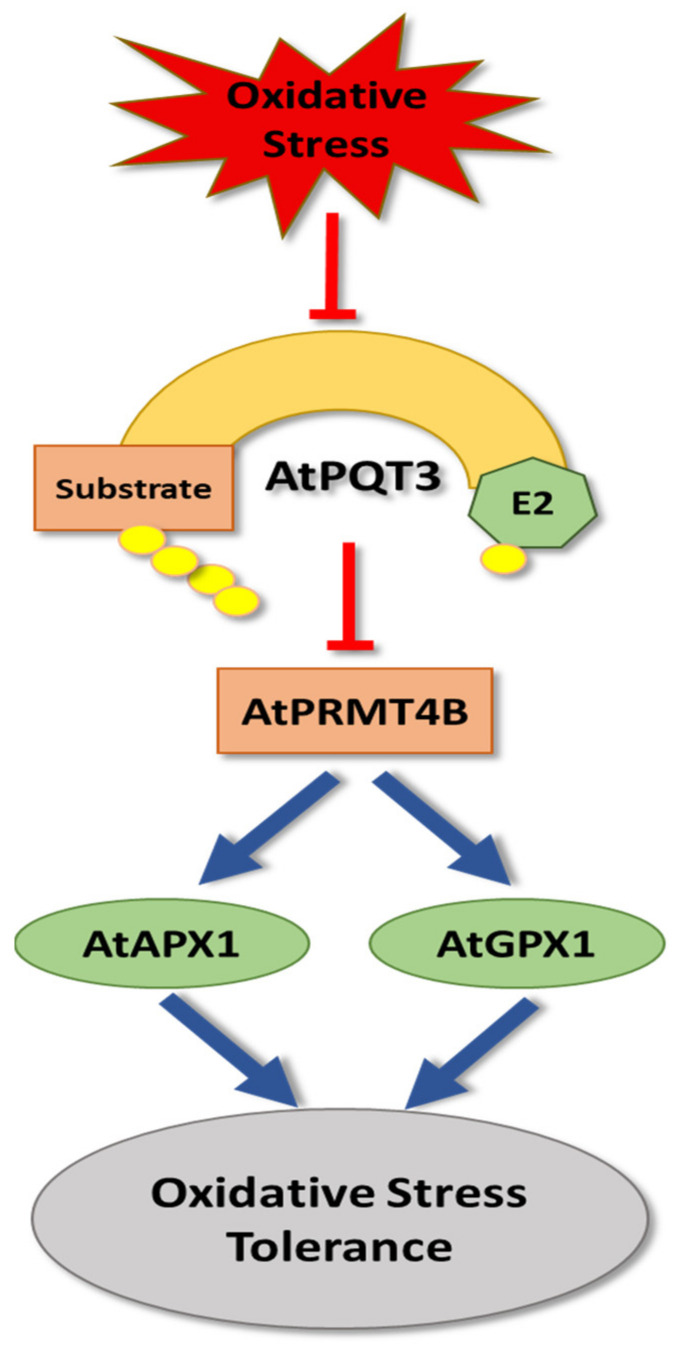
The role of *A. thaliana* PARAQUAT TOLERANCE 3 (PQT3) in oxidative stress tolerance. AtPQT3 negatively regulates oxidative stress tolerance by triggering degradation of PROTEIN ARGININE METHYLTRANSFERASE 4B (AtPRMT4B), which promotes *ASCORBATE PEROXIDASE 1 (APX1) and GLUTATHIONE PEROXIDASE 1 (GPX1)* expression. Both APX1 and GPX1 play major roles in oxidative stress tolerance by acting as antioxidants that catalyze the reduction of H_2_O_2_ to water and oxygen.

Only a few E3 ligases have been described to regulate oxidative stress tolerance (Table 1). For example, Arabidopsis PARAQUAT TOLERANCE 3 (PQT3), a member of RING finger/U-box E3 ligases, has been identified as a major oxidative stress regulator [185] (Figure 5). Protein interaction assays showed that PQT3 directly interacts with PROTEIN ARGININE METHYLTRANSFERASE 4B (AtPRMT4B), which plays a role in upregulating the expression of both *ASCORBATE PEROXIDASE 1 (APX1)* and *GLUTATHIONE PEROXIDASE 1 (GPX1)*. These play major roles in oxidative stress tolerance by acting as antioxidant enzymes to catalyze the reduction of H_2_O_2_ to water and oxygen [186,187]. Under oxidative stress, *PQT3* expression is suppressed, which leads to an accumulation of PRMT4B. PRMT4B, in turn, enhances the histone methylation around *APX1* and *GPX1* gene loci, signaling increases in their expression level [185]. When ROS levels return to normal, PQT3 suppression is released, causing a down regulation of APX1 and GPX1, which characterizes PQT3 as a negative regulator of oxidative stress tolerance [185].

Because the mechanism of action for PQT3 is well understood, this E3 ligase could be a potential candidate for genetic engineering to improve oxidative stress tolerance in crop plants.

In wheat the F-box protein *F-BOX- ANTAGONIST OF MITOTIC EXIT NETWORK PROTEIN 1* (*TaFBA1*) is a subunit of SKP1, CULLIN, F-BOX (SCF) E3 ligase. Expression was up-regulated under oxidative stress treatment, and overexpression of *TaFBA1* in transgenic tobacco plants resulted in increased activities of oxidative stress-related enzymes, such as catalase (CAT), peroxidase (POD), and superoxide dismutase (SOD) [138]. In addition to its role in oxidative stress tolerance, TaFBA1 has been described as a positive regulator of both heat and drought stress tolerance [136,137], making this an interesting candidate for the possible enhancement of yield in wheat under a range of abiotic stresses.

TaPUB1, another example from wheat, has been shown to function as a positive regulator of drought stress tolerance by enhancing cellular antioxidant capacity levels [140]. Under drought stress, transgenic plants that overexpressed *TaPUB1* showed increased CAT and SOD activities, compared to wild type, and accumulated less O_2_^−^ and H_2_O_2_ [140].

The rice U-box E3 ligase OsPUB15 is also involved in oxidative stress tolerance with elevated expression under oxidative stress conditions [130]. *ospub15* mutant lines showed acute growth retardation during seedling establishment, likely corresponding to the observed increase in ROS and oxidized protein levels [130]. On the other hand, transgenic lines that overexpressed *OsPUB15* showed better growth than wild type when exposed to paraquat [130]. These results point out that OsPUB15 acts as a positive regulator of oxidative stress tolerance [130].

Another oxidative stress-related example is the rice STRESS-RELATED RING FINGER PROTEIN 1 (OsSRFP1) [128]. Although *OsSRFP1* transcript levels are induced under H_2_O_2_ treatment, transgenic plants constitutively overexpressing *OsSRFP1* have reduced oxidative stress tolerance, compared to the wild type. *OsSRFP1* knockdowns through RNA interference (RNAi) exhibited an enhanced tolerance towards H_2_O_2_ [128]. Additionally, the RNAi plants had higher levels of both antioxidant enzyme activity and proline pools, indicating that OsSRFP1 serves as a negative regulator of ROS tolerance [128].

Finally, a negative regulator of oxidative stress is the apple (*Malus domestica*) MYB30-INTERACTING E3 LIGASE 1 (MdMIEL1) [160]. Expression levels of *MdMIEL1* are upregulated under H_2_O_2_ treatment. Both transgenic Arabidopsis and apple calli that overexpress *MdMIEL1* displayed a higher level of ROS accumulation along with increased H_2_O_2_ sensitivity [160]. The mechanism of how MdMIEL affects oxidative stress, and its targets, are still unknown [160].

As climate change increases the abiotic stressors that negatively impact plants, oxidative stress will become more relevant. There is still a lot of work to be done to identify the E3 ligases and corresponding substrates that are involved in oxidative stress response. Expanded knowledge in this field could be a useful compliment to further improve resilience under primary abiotic challenges.

## 5. Temperature Stress

In both cases, high or low temperatures can lead to heat or cold stress in plants, and may cause cell death [188,189]. Heat stress generates ROS, which cause damage to macromolecules such as proteins, fats, and nucleic acids [190,191]. They also alter protein structures and affect the structural integrity of membranes due to lipid peroxidation [191]. Cold stress decreases the activity of various enzymes and reduces the fluidity of the plasma membrane, which in turn affects metabolic and physiological processes in the cell [192,193].

Several E3 ligases have been described as regulators of heat stress (Table 1). In Arabidopsis, the PLANT U-BOX 48 (AtPUB48) functions as a positive regulator of heat stress tolerance. Overexpression of *AtPUB48* was connected with significantly greater expression of thermotolerance-related genes and stimulated heat stress tolerance in transgenic plants during the germination and seedling growth stages [94]. Consequently, *atpub48* mutants had lower levels of expression and were hypersensitive to elevated temperatures with significantly decreased germination rates, compared to wild type. This data indicates that AtPUB48 targets transcriptional repressors to allow a proper heat stress response [94].

**Figure 6:**
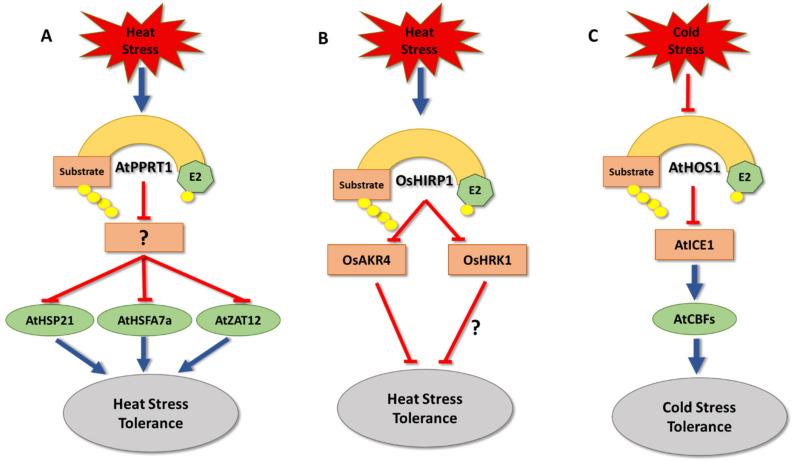
The role of E3 ligases in temperature stress tolerance. (**A**) *Arabidopsis thaliana* PROTEIN WITH THE RING DOMAIN AND TMEMB1 (AtPPRT1) positively regulates heat tolerance by triggering the degradation of unknown substrates, which is likely a negative regulator of HEAT SHOCK PROTEIN 21 (AtHSP21), HEAT SHOCK TRANSCRIPTION FACTOR A7A (AtHSFA7a), and ZINC-FINGER PROTEIN 12 (AtZAT12). The role of *A. thaliana* HIGH EXPRESSION OF OSMOTICALLY RESPONSIVE GENES 1 (HOS1) in cold stress tolerance. (**B**) *Oryza sativa* HEAT-INDUCED RING FINGER PROTEIN 1 (OsHIRP1) positively regulates heat tolerance by triggering degradation of ALDO/KETO REDUCTASE 4 (OsAKR4) and HIRP1-REGULATED KINASE1 (OsHRK1), which is highly likely a negative regulator of heat stress tolerance. (**C**) *Arabidopsis thaliana* HIGH EXPRESSION OF OSMOTICALLY RESPONSIVE GENES 1 (HOS1) negatively regulates cold tolerance by triggering degradation of INDUCER OF CBF EXPRESSION 1 (ICE1), which is a positive regulator of C-REPEAT BINDING FACTORS (CBF) transcription factors. CBFs play major roles in cold stress tolerance by promoting transcription of cold tolerance related genes.

The Arabidopsis RING-finger E3 ligase PROTEIN WITH THE RING DOMAIN AND TMEMB1 (AtPPRT1) is also described to be involved in thermotolerance regulation [106]. *AtPPRT1* transcript levels are increased under heat stress, and *atpprt1* null mutants showed an increase in ROS levels, along with lower seed germination rates, compared to wild type under heat stress [106]. Conversely, seedlings overexpressing *AtPPRT1* became more heat stress tolerant [106], which is likely based on higher expression levels of heat stress-related genes, such as *HEAT SHOCK PROTEIN 21* (*AtHSP21*), *HEAT SHOCK TRANSCRIPTION FACTOR A7A* (*AtHSFA7a*), and *ZINC-FINGER PROTEIN 12* (*AtZAT12*), in comparison to wild type [106,194,195,196]. While these results indicate that AtPPRT1 acts as a positive regulator of thermotolerance, its exact mechanism of action needs further investigation [106] (Figure 6A). In addition to its role in thermotolerance regulation, AtPPRT1 has been reported to be involved in both salt and drought stress tolerances [105,107].

Another positive regulator of high temperature stress response is the rice HEAT-INDUCED RING FINGER PROTEIN 1 (OsHIRP1) [118]. Its expression is increased in plants subjected to higher temperatures, and *OsHIRP1* overexpressing plants gained higher seed germination and seedling survival rates under heat stress conditions, compared to wild type, along with increased expression of heat stress-related genes [118]. Proteomic interaction experiments performed to identify potential targets showed that OsHIRP1 targets at least two proteins for degradation, ALDO/KETO REDUCTASE 4 (OsAKR4) and HIRP1-REGULATED KINASE1 (OsHRK1) [118]. OsAKR4 is known to be involved in thermotolerance regulation, while the function of OsHRK1 is not yet understood [197,198] (Figure 6B).

On the other hand, several E3 ligases have been identified that play roles in regulating cold stress responses (Table 1). For example, in Arabidopsis and rice HIGH EXPRESSION OF OSMOTICALLY RESPONSIVE GENES 1 (HOS1) acts as a negative regulator of cold stress tolerance [97,98,132] by affecting expression of *C-REPEAT BINDING FACTORS* (*CBF*) transcription factors [97,98,132]. CBF transcription factors promote expression of genes that carry *DEHYDRATION RESPONSIVE ELEMENTS* (*DRE*) in their promoter region [199,200,201]. Critical for *CBF* expression is the transcription factor INDUCER OF CBF EXPRESSION 1 (ICE1) [202]. ICE1 has been shown to physically interact with and be ubiquitylated by HOS1, which results in its proteasomal degradation and CBF suppression [98] (Figure 6C).

Similarly in banana (*Musa acuminata*) the RING finger E3 ligase SEVEN IN ABSENTIA (SINA) regulates the stability of MaICE1 [203]. Both protein interaction and ubiquitination assays showed that SINA marks MaICE1 for proteasomal degradation [203], demonstrating that MaSINA, like HOS1 in Arabidopsis and rice, is a negative regulator of cold stress tolerance in banana.

The overall global temperature has been steadily rising over the last decades. This trend is predicted to continue, leading to warmer springs that can impact germination and seedling development. Understanding E3 ligases that positively regulate heat response, such as AtPPRT1 and OsHIRP1, could offer ideas to protect crops during this critical growth phase.

## 6. Heavy Metal Stress

Proteins are basic components of living cells, and they have several important vital roles such as signaling, regulation, structural support, defense, transport, and movement [204]. Structure is a key aspect for proper protein function, and heavy metals (HMs) are one class of harmful chemicals that can disrupt this by forming sulfhydryl bonds. HMs can also displace the normal metals used as enzymatic cofactors, causing functional loss of enzyme activity [205,206,207]. Moreover, an elevation of ROS formation has been identified as a result of antioxidative enzymes being inhibited by HMs [208].

HMs are nondegradable inorganic elements with high density and high atomic mass [208]. Some are considered essential, in minimal amounts, for normal plant growth, (e.g., Cu^2+^, Mo^4+^, Ni^2+^, Se^2+^, Co^2+^, Zn^2+^ and Mn^2+^), while others have no known benefits to plants (e.g., Cd^2+^, Sb^3+^, Cr^3+^, As^3+^, Pb^2+^, Ag^1+^ and Hg^2+^) [209,210]. The high-level uptake of heavy metals in general is harmful [211], and in crops it can cause a reduction in yield [212,213]. There are several E3 ligases that have been described to be involved in the regulation of HMs stress tolerance (Table 1).

In rice HEAVY METAL INDUCED RING E3 LIGASE 1 (OsHIR1) was described as a positive regulator of HMs tolerance [117]. An upregulation of *OsHIR1* transcript levels has been shown under 50 μM Cd^2+^ or 150 μM As^3+^ treatments [117], and Arabidopsis plants overexpressing *OsHIR1* displayed reduced sensitivity toward both Cd^2+^ and As^3+^ exposure. This was likely due to a lower accumulation of these HMs in roots and shoots compared to wildtype [117]. Moreover, it was demonstrated that OsHIR1 interacted with TONOPLAST INTRINSIC PROTEIN 4;1 (OsTIP4;1) and enhanced its degradation via the 26S proteasome pathway [117]. OsTIP4;1 is an aquaporin protein located in the tonoplast and has a high rate of water and glycerol transport [214,215]. Both glycerol and As^3+^ have the same uptake mechanisms, and they compete for transport by OsTIP4;1, which suggests that OsHIR1 decreased the uptake of As^3+^ by promoting the degradation of OsTIP4;1 [117,216].

**Figure 7:**
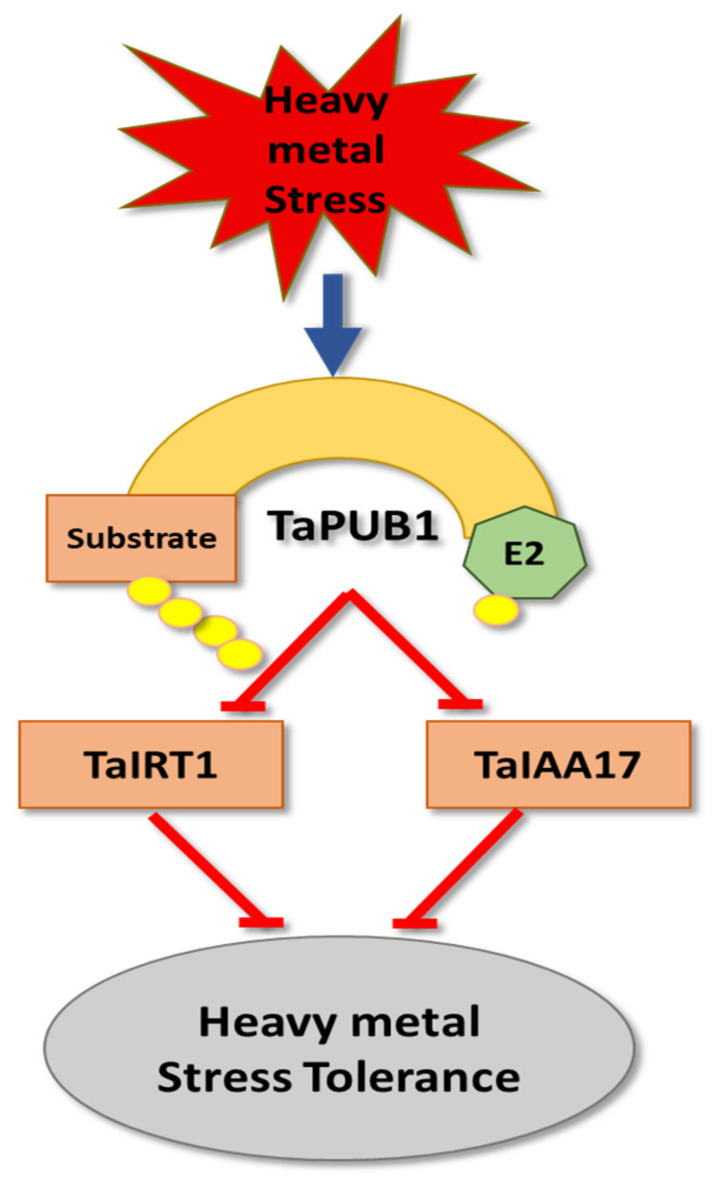
The role of *T. aestivum* PLANT U-BOX 1 (TaPUB1) in heavy metal (HMs) stress tolerance. TaPUB1 positively regulates HM tolerance by triggering degradation of both IRON-REGULATED TRANSPORTER 1 (TaIRT1) and INDOLE-3-ACETIC ACID INDUCIBLE 17 (TaIAA1*7*), which act as negative regulators of HM stress tolerance.

Besides its role as a positive regulator of salt and drought stress in wheat [139,140], the PLANT U-BOX 1 (TaPUB1) was described as a positive regulator for Cd^2+^ stress tolerance [141] (Figure 7). Under Cd^2+^ treatment (200μM CdCl_2_), *TaPUB1* overexpression lines exhibited a reduction in Cd^2+^ accumulation in both roots and shoots and better survival rates and root growth in comparison to wildtype plants. *TaPUB1-RNAi* lines showed opposite findings [141]. TaPUB1 interacts with IRON-REGULATED TRANSPORTER 1 (TaIRT1) and INDOLE-3-ACETIC ACID INDUCIBLE 17 (TaIAA17) and mediates their degradation via the 26S proteasome pathway [141]. TaIRT1 is an iron transporter that facilitates Cd^2+^ uptake. Its accumulation in the root [217,218] suggests that TaPUB1-triggered degradation of TaIRT1 results in the observed reduced Cd^2+^ accumulation [141]. TaIAA17 is a transcriptional regulator that functions as a repressor of auxin-inducible gene expression, and a negative regulator of root elongation growth [219]. Cd^2+^ treatment reduced auxin levels in the plant, stabilized the suppression of auxin signaling by TaIAA17, and reduced root elongation [220,221]. This suggests that TaPUB1 counteracts Cd^2+^-dependent root growth by promoting TaIAA7 degradation [141].

Because TaPUB1 has been reported as a positive regulator of multiple abiotic stresses (drought, salt, and HMs), it could be a potential candidate for genetic engineering research to produce transgenic plants with a broad range of abiotic stress tolerance traits.

In tomato the REALLY INTERESTING NEW GENE 1 (SlRING1) protein is also critical for Cd^2+^ tolerance [157]. Tomato transgenic lines that overexpress *SlRING1* displayed higher photosynthetic rates and higher chlorophyll levels, compared to wildtype, when treated with Cd^2+^ [157]. The transgenic lines also showed a reduction in ROS level and overall membrane damage [157]. Moreover, *SlRING1* overexpressing plants exhibited a decrease in Cd^2+^ accumulation in both roots and shoots, indicating a positive role of SlRING1 in controlling Cd^2+^ tolerance [157].

High levels of HMs in food crops are an especially worrying concern in developing countries [222,223]. In addition to the effects on plant growth, these concentrated HMs are then consumed by people and livestock, which can have unhealthy side effects. Learning more about how E3 ligase activity in plants connected with HM tolerance may help prevent, or at least reduce, the uptake of such contaminants from the soil, providing double benefits for agricultural yield as well as animal and human health.

## 7. Conclusions and Future Perspectives

Abiotic stress is one of the main negative influences that affects the productivity of crops worldwide. Because plants are sessile organisms, they completely depend on fast responsive regulatory mechanisms to withstand stress, which is represented by the UPP, and the diversity of E3 ligases and their substrates. This review highlighted E3 ligases and how critical they are for abiotic stress tolerance, but it also becomes clear how little is known about the regulatory mechanisms and substrate proteins targeted in order to cope with detrimental situations. For example, in Arabidopsis, one of the first plants fully sequenced, more than 1100 potential E3 ligases have been discovered to date. However, for most, their biological roles are still unknown, and their predicted role as E3 ligases is normally only based on the presence of certain motifs, such as RING or U-box [12,13].

It will be critical in the future to systematically investigate the remaining E3 ligase candidates, whether they are indeed active within the UPP, and to what extent they affect abiotic stress responses. It is expected that this will still require step-by-step analysis of individual E3 ligases using classical gain- and loss-of-function approaches. However, modern technologies that utilize the powers of proteomics and transcriptomics will be needed in the future to effectively unravel the breadth of substrates and the impacts specific E3 ligases have on cellular systems. Specific tools that could be useful include protein-protein interaction assays that can detect the moderate and weak transient interactions, such as protein microarrays, which support analyzing thousands of proteins at the same time in a single experiment, and provide instant detection of two protein interactions. Both bimolecular fluorescence complementation (BiFC) and split-luciferase assay (Split-LUC) can be used to provide direct information of protein-protein interaction and subcellular localization of the interaction in living plant cells [224]. Such data will help to develop detailed models of abiotic stress responses and clarify the roles that E3 ligases play in plants, which will facilitate the design of novel approaches to generate more stress resilient crops in response to global changes in farming conditions.

## Figures and Tables

**Table 1:** Plant E3 ligases involved in abiotic stress tolerance.

Species	Name	Kind	Abiotic Stress Function	Citation
*Arabidopsis*	RGLG1 RGLG2 RING DOMAIN LIGASE	RING finger domain E3 ubiquitin ligase	Negatively regulates drought stress tolerance	[76]
	DOR DROUGHT TOLERANCE REPRESSOR	Skp1–Cullin–F-box (SCF) RING finger E3 ligase	Negatively regulates ABA-dependent drought tolerance	[77]
	AFA1 ARABIDOPSIS F-BOX PROTEIN HYPERSENSITIVE TO ABA 1	Skp1–Cullin–F-box (SCF) RING finger E3 ligase	Negatively regulates ABA-dependent drought tolerance	[78]
	SDR SALT AND DROUGHT RESPONSIVENESS	Skp1–Cullin–F-box (SCF) RING finger E3 ligase	Positively regulate salt stress tolerance, negatively regulate drought stress tolerance	[79]
	SAP5 STRESS ASSOCIATED PROTEIN 5	RING finger domain E3 ubiquitin ligase	Positively regulates salt, drought, and osmotic stress tolerance	[80]
	AIRP1 AIRP2 ABA-INSENSITIVE RING PROTEIN	C3H2C3-type RING finger E3 ubiquitin ligase	Positively regulates ABA-dependent drought tolerance	[81,82]
	AIRP3/LOG2 ABA-INSENSITIVE RING PROTEIN3/LOSS OF GLUTAMINE DUMPER 2	RING finger domain E3 ubiquitin ligase	Positively regulates the ABA-mediated drought and salt stress tolerance	[83]
	ATRF1 ALUMINUM TOLERANCE RING FINGER 1	C3H2C3-type RING finger E3 ubiquitin ligase	Positively regulates aluminum tolerance	[84]
	NERF NFYA5 ENHANCING RING FINGER	RING finger domain E3 ubiquitin ligase	Positively regulates drought stress response	[85]
	RDUF1 RDUF2 RING DOMAIN OF UNKNOWN FUNCTION	RING finger domain E3 ubiquitin ligase	Positively regulates salt stress responses and ABA-dependent drought stress responses in arabidopsis	[84,86]
	PQ3 PARAQUAT TOLERANCE 3	U-box E3 ubiquitin ligase	Negatively regulates oxidative stress response	[87]
	PP2-B11 PHLOEM PROTEIN 2-B11	Skp1–Cullin–F-box (SCF) RING finger E3 ligase	Negatively regulates drought stress and positively regulate salt stress	[88,89]
	PUB11 PLANT U-BOX 11	U-box E3 ubiquitin ligase	Negatively regulates drought tolerance	[67]
	PUB19 PLANT U-BOX 19	U-box E3 ubiquitin ligase	Negatively regulates drought stress tolerance	[90]
	PUB22 PUB23 PLANT U-BOX	U-box E3 ubiquitin ligase	Negatively regulates drought stress tolerance	[66]
	PUB30 PLANT U-BOX 30	U-box E3 ubiquitin ligase	Negatively regulates the salt stress tolerance during germination	[91]
	PUB46 PLANT U-BOX	U-box E3 ubiquitin ligase	Positively regulates oxidative stress tolerance	[92]
	PUB46 PUB48 PLANT U-BOX	U-box E3 ubiquitin ligase	Positively regulate drought stress tolerance	[93]
	PUB48 PLANT U-BOX 48	U-box E3 ubiquitin ligase	Positively regulates heat tolerance	[94]
	SDIR1 SALT- AND DROUGHT-INDUCED RING FINGER1	RING finger domain E3 ubiquitin ligase	Positively regulates ABA dependent salt and drought stress tolerance	[95]
	PRU1 PHOSPHATE RESPONSE UBIQUITIN E3 LIGASE1	RING finger domain E3 ubiquitin ligase	Modulates Pi homeostasis in response to low-Pi stress in Arabidopsis	[96]
	HOS1 HIGH EXPRESSION OF OSMOTICALLY RESPONSIVE GENE 1	RING finger domain E3 ubiquitin ligase	Negatively regulates the cold stress response in Arabidopsis	[97,98]
	DUF1 DUF2 DOMAIN OF UNKNOWN FUNCTION	RING finger domain E3 ubiquitin ligase	Positively regulates ABA dependent drought stress response	[99]
	ATL61 ATL78 ARABIDOPSIS TÓXICOS EN LEVADURA	C3H2C3 RING finger domain E3 ubiquitin ligase	Negatively regulates cold stress response and a positively regulates drought stress response	[100,101]
	STRF1 SALT TOLERANCE RING FINGER 1	RING finger domain E3 ubiquitin ligase	Positively regulates salt stress	[102]
	JUL1 JAV1-ASSOCIATED UBIQUITIN LIGASE1	C3H2C3 RING finger domain E3 ubiquitin ligase	Positively regulates drought stress	[103]
	SR1 SUBMERGENCE RESISTANT1	RING finger domain E3 ubiquitin ligase	Negatively regulates submergence tolerance	[104]
	PPRT1 PROTEIN WITH THE RING DOMAIN AND TMEMB 1	C3H2C3 RING finger domain E3 ubiquitin ligase	Negatively regulates salt stress response. positive role in regulating the high temperature. Negative role in ABA and drought stress responses	[105,106,107]
	RHA2B RHA2A RING-H2 FINGER PROTEIN 2B/2A	RING finger domain E3 ubiquitin ligase	Positively regulates ABA-dependent drought response	[108,109]
	RZP34/CHYR1 RING ZINC-FINGER PROTEIN34/CHY ZINC-FINGER AND RING PROTEIN1	RING finger domain E3 ubiquitin ligase	Positively regulates drought stress tolerant	[110]
	XBAT35.2 XB3 ORTHOLOG 5 IN ARABIDOPSIS THALIANA	RING finger domain E3 ubiquitin ligase	Negatively regulates the drought and salt stress response	[111]
Rice *Oryza sativa*	AIR4.1 AIR4.2 ARSENIC-INDUCED RING E3 LIGASE4	RING finger domain E3 ubiquitin ligase	Positively regulates Arsenic stress tolerance	[112]
	CBE1 CULLIN4-BASED E3 UBIQUITIN LIGASE1	Cullin4-Based E3 Ubiquitin Ligase	Negatively regulates abiotic stress tolerance	[113]
	DSG1 DELAYED SEED GERMINATION 1	RING finger domain E3 ubiquitin ligase	Negatively regulates salt and drought stress tolerance	[114]
	RDCPS RING DOMAIN-CONTAINING PROTEINS	RING finger domain E3 ubiquitin ligase	Positively regulates drought stress tolerant	[115]
	DIS1 DROUGHT-INDUCED SINA PROTEIN 1	C3HC4 RING finger domain E3 ubiquitin ligase	Negatively Regulates drought response	[116]
	HIR1 HEAVY METAL INDUCED RING E3 LIGASE 1	RING finger domain E3 ubiquitin ligase	Positively regulates heavy metal tolerance	[117]
	HIRP1 HEAT INDUCED RING FINGER PROTEIN 1	RING finger domain E3 ubiquitin ligase	Positively regulates plant response to heat stress	[118]
	MAR1 MICROTUBULE-ASSOCIATED RING FINGER PROTEIN 1	RING finger domain E3 ubiquitin ligase	Negatively regulates the salt-stress response	[119]
	RHP1 RING-H2 FINGER PROTEIN 1	RING finger domain E3 ubiquitin ligase	Positively regulates salt and drought stress tolerance	[120]
	SDIR1 SALT-AND DROUGHT-INDUCED RING FINGER 1	RING finger domain E3 ubiquitin ligase	Positively regulates drought stress tolerance	[121]
	SIRF1 SALT INDUCED RING FINGER PROTEIN	RING finger domain E3 ubiquitin ligase	Positively regulates salt and osmotic stress	[122]
	SIRP1 SALT-INDUCED RING PROTEIN 1	RING finger domain E3 ubiquitin ligase	Negatively regulates of salinity stress tolerance	[123]
	SIRP2 SALT-INDUCED RING PROTEIN 2	RING finger domain E3 ubiquitin ligase	Positively regulates of salt and osmotic stress tolerance	[124]
	SIRP3 SALT-INDUCED RING PROTEIN 3	RING finger domain E3 ubiquitin ligase	Negatively regulates salinity stress response	[125]
	SIRP4 SALT-INDUCED RING PROTEIN 4	RING finger domain E3 ubiquitin ligase	Negatively regulates salt stress responses	[126]
	SIRH2- 14 SALT-INSENSITIVE RING-H2 TYPE 14	RING finger domain E3 ubiquitin ligase	Positively regulates salt stress tolerance	[127]
	SRFP1 STRESS-RELATED RING FINGER PROTEIN 1	RING finger domain E3 ubiquitin ligase	Negatively regulates salt, cold and oxidative stresses	[128]
	PUB2 PUB3 PLANT U-BOX 2AND 3	U-box E3 ubiquitin ligase	Positively regulates the response to cold stress	[129]
	PUB15 PLANT U-BOX 15	U-box E3 ubiquitin ligase	Positively regulates oxidative stress tolerance	[130]
	PUB41 PLANT U-BOX41	U-box E3 ubiquitin ligase	Negatively regulates drought stress response	[65]
	SADR1 SALT, ABA, AND DROUGHT STRESS-INDUCED RING FINGER PROTEIN 1	RING finger domain E3 ubiquitin ligase	Negatively Regulates Response to Salinity and drought stress	[131]
	HOS1 HIGH EXPRESSION OF OSMOTICALLY RESPONSIVE GENES 1	RING finger domain E3 ubiquitin ligase	Negatively regulates the cold stress response	[132]
	DIRP1 DROUGHT-INDUCED RING PROTEIN 1	RING finger domain E3 ubiquitin ligase	Negatively regulates drought and salt stress, and positively regulates cold stress response in rice	[133]
	DHSRP1 DROUGHT-HEAT-SALT INDUCED RING FINGER PROTEIN 1	RING finger domain E3 ubiquitin ligase	Negatively regulates drought, heat, and salt stress tolerance	[134]
Wheat *Triticum aestivum*	DIS1 DROUGHT-INDUCED SINA PROTEIN 1	C3HC4 RING finger domain E3 ubiquitin ligase	Negatively regulates drought stress tolerance	[135]
	FBA1 FBOX-AMN1	Skp1–Cullin–F-box (SCF) RING finger E3 ligase	Positively regulates heat and drought stress in wheat. Positively regulates oxidative stress	[136,137,138]
	PUB1 PLANT U-BOX 1	U-box E3 ubiquitin ligase	Positively regulates salt and drought stress tolerance in wheat. Positively regulate Cadmium stress tolerance	[139,140,141]
	PUB15 PLANT U-BOX 15	U-box E3 ubiquitin ligase	Positively regulates salt stress tolerance in wheat	[142]
	PUB26 PLANT U-BOX 26	U-box E3 ubiquitin ligase	Negatively regulates salt stress in wheat	[143]
	SAP5 STRESS-ASSOCIATED PROTEIN	RING finger domain E3 ubiquitin ligase	Positively regulates drought stress	[144]
	ZNF ZINC-FINGER PROTEIN	C3HC4 RING finger domain E3 ubiquitin ligase	Positively regulates salt and drought stress	[145]
Pepper *Capsicum annuum*	ASRF1 ABA SENSITIVE RING FINGER E3 LIGASE 1	RING finger domain E3 ubiquitin ligase	Positively regulates drought stress tolerance in pepper	[146]
	AIRE1 ABA INDUCED RING-TYPE E3 LIGASE 1	RING finger domain E3 ubiquitin ligase	Positively regulates the drought stress response in pepper	[147]
	AIR1 ABA-INSENSITIVE RING PROTEIN 1 GENE	RING finger domain E3 ubiquitin ligase	Negatively regulates the ABA-mediated drought stress tolerance mechanism	[148]
	DTR1 DROUGHT TOLERANCE RING 1	RING finger domain E3 ubiquitin ligase	Positively regulates the drought stress response in pepper	[149]
	ATIR1 ATBZ1 INTERACTING RING FINGER PROTEIN 1	RING finger domain E3 ubiquitin ligase	Positively regulates abscisic acid signaling and drought response	[150]
	REL1 RING TYPE E3 LIGASE 1 GENE	RING finger domain E3 ubiquitin ligase	Negatively regulates ABA-mediated drought stress tolerance	[151]
	DIR1 DROUGHT INDUCED RING TYPE E3 LIGASE 1	RING finger domain E3 ubiquitin ligase	Negatively regulates the drought stress response via ABA-mediated signaling	[152]
	AIRF1 ADIP1 INTERACTING RING FINGER PROTEIN 1	RING finger domain E3 ubiquitin ligase	Positively regulates the drought stress response in pepper	[153]
Corn *Zea maiz*	AIRP4: *ZEA MAYS* ABA INSENSITIVE RING PROTEIN 4	RING finger domain E3 ubiquitin ligase	Positively regulates the drought tolerance response pathway	[154]
	RFP1 RING FINGER PROTEIN 1	RING finger domain E3 ubiquitin ligase	Positively regulates salt and drought stress tolerance	[155]
Cotton *Gossypium hirsutum*	SARP1 SALT-ASSOCIATED RING FINGER PROTEIN	C3H2C3 RING finger domain E3 ubiquitin ligase	Negatively regulate the response to salt stress	[156]
Tomato *Solanum lycopersicum*	RING1REALLY INTERESTING NEW GENE 1	RING finger domain E3 ubiquitin ligase	Positively regulates Cd tolerance	[157]
Sickle medick *Medicago falcata*	STMIR SALT TUNICAMYCIN-INDUCED RING FINGER PROTEIN	RING finger domain E3 ubiquitin ligase	Positively regulates salt stress	[158]
Wild tomato *Solanum pimpinellifolium*	RING REALLY INTERESTING NEW GENE	RING finger domain E3 ubiquitin ligase	Positively regulates salt stress in wild tomato species	[159]
Apple *Malus domestica*	MIEL MYB30-INTERACTING E3 LIGASE 1	RING finger domain E3 ubiquitin ligase	Negatively regulates salt and oxidative stresses tolerance	[160]
Soybean *Glycine max*	PUB6 PUB8 PLANT U-BOX	U-box E3 ubiquitin ligase	Negative regulator of drought stress response in Arabidopsis	[161,162]

## Abbreviations

ABC1P11	ABC domain containing protein
AKR4	aldo/keto reductase 4
APX1	ascorbate peroxidase 1
BiFC	bimolecular fluorescence complementation
BTB/POZ	broad-complex, tramtrack, and bric-à-brac/poxvirus and zinc finger
CAT	catalase
CBF	C-repeat binding factors
CLC 6	chloride channel 6
CRL3	cullin3 ring e3 ligases
CRL4	cullin4 ring e3 ligases
DOR	drought tolerance repressor
DRE	dehydration responsive elements
DREB2A	dehydration-responsive element binding protein2a
DRIP1	DREB2A interacting protein1
ERF53	ethylene response factor53
FBA1	F-box- antagonist of mitotic exit network protein 1
GPX1	glutathione peroxidase 1
HECT	homology to the E6-associated protein c-terminus
HIR1	heavy metal induced ring e3 ligase 1
HIRP1	heat-induced ring finger protein 1
HMS	heavy metals
HOS1	high expression of osmotically responsive genes 1
HRK1	hirp1-regulated kinase1
HSFA7A	heat shock transcription factor a7a
HSP21	heat shock protein 21
IAA17	indole-3-acetic acid inducible 17
ICE1	inducer of cbf expression 1
IRT1	iron-regulated transporter 1
KIN7	kinase 7
LRR1	leucine rich repeat protein 1
MADS70	mads-box gene 70
MAPKKK18	mitogen activated protein kinase kinase kinase 18
MIEL1	myb30-interacting e3 ligase 1
PEX11-1	peroxisomal biogenesis factor 11-1
POD	Peroxidase
PP2CA	protein phosphatase 2ca
PPRT1	protein with the ring domain and tmemb1
PQT3	paraquat tolerance 3
PRMT4B	protein arginine methyltransferase 4b
PUB	plant u box
RGLG1	ring domain ligase1
RING	really interesting new gene
ROS	reactive oxygen species
RPT2A	regulatory particle aaa-atpase 2a
SAP5	stress-associated protein 5
SARP1	salt-associated ring finger protein
SCF	skip1-cullin1-f-box
SINA	seven in absentia
SIRP	salt-induced ring protein
SOD	superoxide dismutase
Split-LUC	Split-luciferase assay
SRFP1	stress-related ring finger protein 1
STRF1	salt tolerance ring finger 1
TIP4;1	tonoplast intrinsic protein 4;1
TKL1	transketolase 1
UBQ	Ubiquitin
UPP	ubiquitin proteasome pathway
ZAT12	zinc-finger protein 12
